# Oral Treatment with the Extract of *Euterpe oleracea* Mart. Improves Motor Dysfunction and Reduces Brain Injury in Rats Subjected to Ischemic Stroke

**DOI:** 10.3390/nu15051207

**Published:** 2023-02-28

**Authors:** Leonan Lima Teixeira, Helma Maria Negrão da Silva Alencar, Luan Oliveira Ferreira, João Cleiton Martins Rodrigues, Rafael Dias de Souza, Laine Celestino Pinto, Nilton Akio Muto, Hervé Rogez, Arnaldo Jorge Martins-Filho, Vanessa Joia de Mello, Moises Hamoy, Edmar Tavares da Costa, Dielly Catrina Favacho Lopes

**Affiliations:** 1 Laboratory of Experimental Neuropathology, João de Barros Barreto University Hospital, Federal University of Pará, Belém 66073-000, Brazil; 2 Centre for the Valorization of Amazonian Bioactive Compounds (CVACBA), Federal University of Pará, Belém 66075-110, Brazil; 3 Department of Pathology, Evandro Chagas Institute, Ananindeua 67030-000, Brazil; 4 Laboratory of Pharmacology and Toxicology of Natural Products, Institute Biological Science, Federal University of Pará, Belém 66077-830, Brazil

**Keywords:** *Euterpe oleracea*, stroke, brain injury, neuroprotection, açaí

## Abstract

Ischemic stroke is one of the principal causes of morbidity and mortality around the world. The pathophysiological mechanisms that lead to the formation of the stroke lesions range from the bioenergetic failure of the cells and the intense production of reactive oxygen species to neuroinflammation. The fruit of the açaí palm, *Euterpe oleracea* Mart. (EO), is consumed by traditional populations in the Brazilian Amazon region, and it is known to have antioxidant and anti-inflammatory properties. We evaluated whether the clarified extract of EO was capable of reducing the area of lesion and promoting neuronal survival following ischemic stroke in rats. Animals submitted to ischemic stroke and treated with EO extract presented a significant improvement in their neurological deficit from the ninth day onward. We also observed a reduction in the extent of the cerebral injury and the preservation of the neurons of the cortical layers. Taken together, our findings indicate that treatment with EO extract in the acute phase following a stroke can trigger signaling pathways that culminate in neuronal survival and promote the partial recovery of neurological scores. However, further detailed studies of the intracellular signaling pathways are needed to better understand the mechanisms involved.

## 1. Introduction

Stroke is the second leading cause of death in the world and the primary cause of disability [1] resulting in a loss of quality of life [2] and a significant burden for national and regional health systems. Ischemic stroke involves cellular and molecular events that begin with the bioenergetic failure of cells by processes that include global or focal brain hypoperfusion, excitotoxicity, oxidative stress, neuro-inflammation, blood-brain barrier dysfunction, and microvascular injuries which culminate in the death of neurons, as well as glial and endothelial cells [3,4,5].

While it is necessary to reverse ischemic stroke, tissue reperfusion can worsen the injuries by overloading the reactive oxygen species during the restoration of the blood flow [4]. In this context, many recent studies have focused on the antioxidant and anti-inflammatory properties of natural products in search of complementary therapeutic strategies for the attention of ischemia-reperfusion injuries [6,7].

The açaí palm, *Euterpe oleracea* Mart. (family Arecaceae), is widely distributed in the Brazilian Amazon region and neighboring areas of Guyana, French Guyana, Surinam, Venezuela, Colombia, Ecuador, and Panama. This palm is found in both seasonally-flooded swamps (known in Brazil as *várzeas*) and on higher ground in areas with high precipitation rates [8,9]. The fruit (pericarp) is rich in proteins, fibers, lipids, and saturated and unsaturated fatty acids [10,11,12].

A number of recent studies have shown that the extracts of açaí fruit, leaves, and roots, and the oil of the fruit are rich in substances with antioxidant and anti-inflammatory properties that have also exhibited other biological activities, including antinociceptive, antimicrobial, and anticonvulsant properties [13,14,15,16,17,18,19,20]. These biological effects may be related to the chemical composition of the açaí fruit which is known to contain high concentrations of polyphenolic compounds such as anthocyanins and tocopherols, with the most abundant substances being cyanidin-glucoside, cyanidin-rutinoside, peonidin-glycoside, epicatechin, catechin, homoorientin, orientin, vitexin, isovitexin, taxifolin, and ferulic acid [14,21,22,23]. Polyphenolic compounds, such as flavonoids and proanthocyanidins, are widely reported in the literature to decrease the harmful effects of inflammatory processes, in addition to their antioxidant activity to reduce mainly reactive oxygen species [24].

Based on its known ethnopharmacological properties and potential benefits for human health, and given its growing relevance in human diets, the present study investigated the potential effects of açaí on ischemic events in a murine model of middle cerebral artery occlusion (MCAO). The study also evaluated the influence of the treatment on the histopathology of the area of ischemic lesion and neuronal death, on biochemical parameters, and on motor and behavioral alterations.

## 2. Materials and Methods

### 2.1. Clarified Lyophilized Açaí (Euterpe oleracea) Extract

The clarified lyophilized extract of *Euterpe oleracea* Mart. (EO) was kindly provided by Amazon Dreams (Belém, Pará, Brazil). The extract is obtained from the fruit (pericarp) using a production process that is patented by Amazon Dreams in collaboration with the Federal University of Pará (PI 8 1003060-3). The phytochemical procedures and the validation of the UHPLC-DAD methods have been described previously, and the analyses have shown that the principal compounds found in the fruit are cyanidin-3-glucoside (180 mg/L), cyanidin-3-rutinoside (450 mg/L), homoorientin (250 mg/L), orientin (380 mg/L), and taxifolin (310 mg/L) [14,20,25,26].

The work extract was reconstituted in filtered water to a concentration of 200 mg/mL, while the dosage used in the present study—200 mg/kg/day—was based on the findings of previous research [27,28].

### 2.2. Animals and Experimental Design

All the experimental procedures were conducted in accordance with the principles of the Brazilian National Council for the Control of Animal Experimentation (CONCEA) and were approved by the Ethics Committee on the Use of Animals of the Biological Sciences Institute of the Federal University of Pará (ICB-UFPA; CEUA No. 1225030320). All necessary precautions were taken to prevent animal suffering and distress.

For the present study, 36 adult (8–10 weeks) male Wistar rats weighing 220 g (±30 g) were obtained from the Central Animal Facility of the Biological Sciences Institute of Federal University of Pará (ICB-UFPA). These animals were housed in polypropylene cages covered by a metallic grid in a controlled environment (22 ± 2 °C; 12/12 h light/dark cycle) with ad libitum access to standard rat chow and water.

After a seven-day acclimation period, the animals were allocated randomly to one of four experimental groups (*n* = 9 animals/group): (i) sham, (negative control), in which the animals received only the vehicle (water); (ii) sham + EO (positive control), in which the animals received the *Euterpe oleracea* (EO) extract; (iii) MCAO, the animals were submitted to middle cerebral artery occlusion for 30 min and received only the vehicle; or (iv) MCAO + EO. The surgical procedures were conducted invariably between 08:00 a.m. and 11:00 a.m.

**Figure 1:**
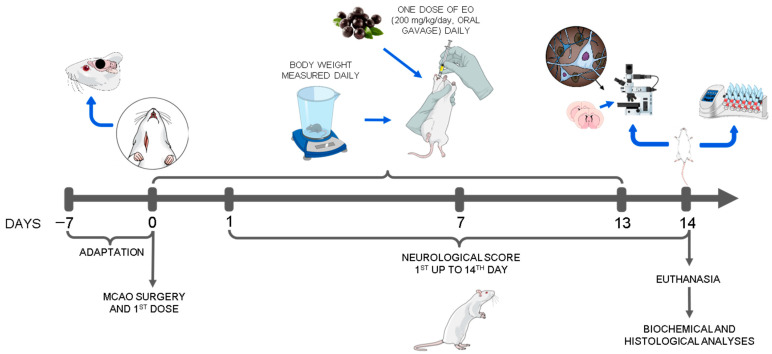
Experimental design using the animals submitted to middle cerebral artery occlusion (MCAO) and treated with clarified extract of *Euterpe oleracea* (EO).

These four groups were monitored for 14 days after the surgery. The MCAO + EO animals received a dose of EO extract (200 mg/kg/day) via oral gavage 4.5 h after the beginning of the MCAO and an additional dose every day until the end of the experiment. Neurological scores were obtained every day during the experiment, with the first evaluation taking place 24 h after the first application of the EO extract, and the last record being obtained on the day following the final application. Neurological tests were conducted on the seventh and fourteenth days after the MCAO. Prior to euthanasia, blood samples were collected via cardiac puncture for biochemical analysis. After euthanasia, the brains were extracted, sectioned, and stained with cresyl violet (0.3%) and neuronal antibody (Figure 1).

### 2.3. Middle Cerebral Artery Occlusion Surgery

The MCAO surgery was conducted as described by Ferreira et al. [29]. For this process, the animals were anesthetized intraperitoneally (i.p.) with ketamine (80 mg/kg) and xylazine (10 mg/kg), then placed on a heated blanket. After the abolishment of the corneal reflex, the common carotid bifurcation was exposed through an incision to the cervical midline, revealing the internal and external carotid arteries. A silicone-coated nylon monofilament (Doccol Corp., Redlands, CA, USA) was then inserted into the stump of the external carotid artery and fed up the internal carotid artery to occlude the origin of the middle cerebral artery. The filament was withdrawn after 30 min to allow for reperfusion. The sham-operated animals were anesthetized and the carotid bifurcation exposed, but no filament was inserted. The incision was sutured, and the animals were returned to their home cages to regain consciousness, receiving dipyrone (500 mg/kg) subcutaneously for analgesia. All the sham-operated rats survived, although mortality was 33.3% in the MCAO group (3 of the 9 animals), and 11.1% (one animal) in the MCAO + EO group.

### 2.4. Neurological Score and Behavioral Test

The neurological motor deficit (clinical scores) of the rats was evaluated daily after the MCAO, with the first score being recorded 24 h after first application of the EO extract and the final score being obtained on the day after the last application of the extract. Neurological deficit was assessed based on the five-point scale of Zhang et al. [30]: 0 = no neurological deficit; 1 = mild loss of contralateral forelimb muscle tone; 2 = loss of muscle tone in the contralateral forelimb of the lesion and circular movement to the side contralateral to the lesion when suspended by the tail; 3 = spontaneous circular movement to the contralateral side of the lesion; 4 = no spontaneous motor activity. The animals with a score of 4 were euthanized to avoid suffering and distress. The observer that evaluated the rats was blind to the treatments.

### 2.5. Histological Analyses

After euthanasia, the animals were perfused transcardially with phosphate buffer saline (PBS, pH 7.4; 4 °C) and then with 4% formaldehyde (pH 7.4). The brain was then extracted from the skull and fixed in 4% formaldehyde for 72 h, followed by cryoprotection in 30% sucrose for a further 72 h before being cut into serial coronal sections (40 μm) in a Leica 1850 UV semi-automatic cryostat (Leica Microsystems, Wetzlar, Germany).

The sections were mounted on gelatin-coated microscope slides, air-dried, and then stained with cresyl violet (0.3%) to measure the percentage area of cerebral infarction [31]. The sections were then dehydrated in an increasing ethanol series, cleared in xylene, and coverslipped with Entellan (Merck, Danstadt, Germany). The images were captured using a Toshiba digital camera (Toshiba America Inc., NY, USA), and the percentage of infarction area (PIA) was calculated using the formula [29]: PIA = (area of infarction/area of the ipsilateral hemisphere) × 100.

The neuronal cells were immunostained overnight at 4 °C using the free-floating method with the anti-NeuN mouse primary antibody (1:2000, Merck-Millipore, Darmstadt, Germany). After washing with phosphate buffered saline (PBS), a DAKO EnVisionTM + Dual Link System-HRP kit (Carpinteria, CA, USA) was used according to the manufacturer’s protocol. This system is based on an HRP-labeled polymer which is conjugated with secondary antibodies. After washing with PBS, DAB (3,3′-diaminobendine; Sigma-Aldrich) was used as a chromogen to stain the sections. Finally, the sections were mounted on gelatin-coated microscope slides, air-dried, dehydrated in an increasing ethanol series, cleared in xylene, and coverslipped with DPX mounting medium. The images were captured using a Toshiba digital camera (Toshiba America Inc., NY, USA) and analyzed with the Stereoinvestigator (MBF Bioscience, Williston, VT, USA) and ImageJ software (NIH, Bethesda, MD, USA). The NeuN^+^ cells were counted in eight sections from the lesion and peri-infarction area in the somatosensory cortex, and the striatum ipsilateral to the area of the lesion in sample fields of 200 μm × 200 μm, separated by cortical layers, with *n* = 6 animals per group [32].

### 2.6. Biochemical Analysis

For the biochemical assays, the serum was extracted from the blood samples (obtained by cardiac puncture) by centrifugation at 3000 rpm for 10 min. The following nine biochemical parameters were determined using a Wiener CM200 chemical analyzer, following the manufacturer’s instructions: aspartate aminotransferase (AST), alanine aminotransferase (ALT), urea (URE), creatinine (CRE), high-density lipoprotein (HDL), very low-density lipoprotein (VLDL), low-density lipoprotein (LDL), total cholesterol (CHO), and triglyceride (TRY). All the analyses were run in the ICB-UFPA Laboratory of Clinical Analyses (LAC).

### 2.7. Statistical Analyses

Prior to the analyses, the normality of the variances was verified using the Kolmogorov-Smirnov test. The data are presented as the mean with the standard deviation (SD), and the *F* and *p* values included whenever relevant. A *p* < 0.05 significance level was considered for all the analyses. Differences between pairs of groups were analyzed using Student’s *t* test, while those among three or more groups were evaluated using a two-way Analysis of Variance (ANOVA), followed by Tukey’s post hoc test for pairwise comparisons. The data were analyzed using GraphPad Prism, version 9 (Graph-Pad Software Inc., San Diego, CA, USA).

## 3. Results

### 3.1. Açaí Extract Improves the Behavioral Outcome of Animals Submitted to Ischemic Stroke

**Figure 2:**
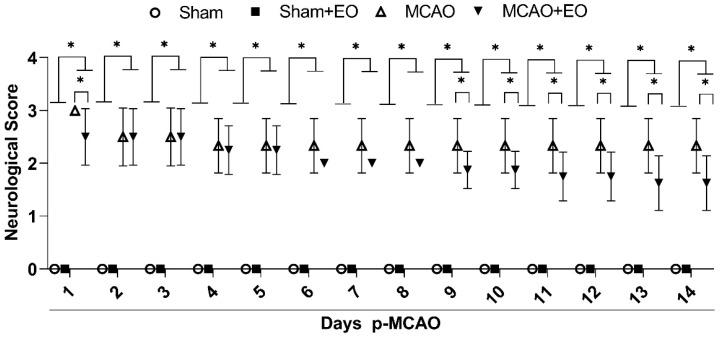
Neurological/motor deficit scores of the animals submitted to middle cerebral artery occlusion (MCAO) and treated with clarified *Euterpe oleracea* extract. The data are presented as the mean ± SD (*n* = 6–9 animals/group; two-way ANOVA followed by Tukey’s post hoc test; ∗ *p* < 0.05).

The neurological scores varied significantly among treatment groups and days (F _(39, 392)_ = 2.419; *p* < 0.0001; Figure 2). The comparison of the days among treatment groups (F _(3, 392)_ = 1996; *p* < 0.0001) showed that there was no significant variation between the sham and sham + EO groups (control groups) over the course of the experiment (*p* > 0.9999). The neurological scores of the ischemic animals (MCAO groups) were significantly different from those of the control groups (*p* < 0.0001, for all comparisons). The animals of the MCAO group presented high rates of spontaneous circular movement to the contralateral side of the lesion (score 3), while treatment with EO extract (MCAO + EO group) alleviated the neurological dysfunction significantly in comparison with the MCAO group from the ninth day (D9) onward (D9 and D10: *p* = 0.0287; D11 and D12: *p* = 0.0021; D13 and D14: *p* < 0.0001; for MCAO vs. MCAO + EO groups), with the rats presenting a loss of muscle tone in the contralateral forelimb and circular movements to the contralateral side when suspended by the tail (score 2).

Clinical improvement was observed in the MCAO + EO group during the course of the treatment (Supplementary Table S1), with days D9 and D10 being significantly different in comparison with D1 and D2 (*p* < 0.01), and D11–D12 significantly different from D1–D3 (*p* < 0.01). Days D13–D14 were also significantly different from each of the five first days of the treatment, i.e., D1–D5 (*p* < 0.01).

### 3.2. Açaí Extract Reduces the Size of the Area of Infarction and Increases the Number of Surviving Neurons after Ischemic Stroke

**Figure 3:**
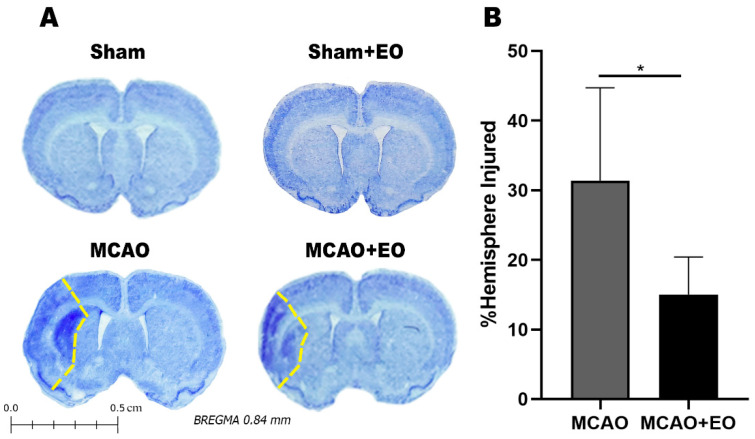
Injured area and percentage of cerebral infarction of animals submitted to middle cerebral artery occlusion (MCAO) and treated with clarified *Euterpe oleracea* extract: (**A**) Representative image of an injured hemisphere; (**B**) Mean percentage of injury in the cerebral hemisphere. The data are expressed as the mean ± SD (*n* = 6 animals per group; Student’s *t* test; * *p* < 0.05).

The behavioral changes observed in the ischemic animals are consistent with lesions in the cortical and subcortical regions and indicate attenuation of the damage by treatment with the EO extract. The ischemic animals presented cortical-subcortical injuries with damage in the primary and secondary somatosensory areas and in the striatum, with a mean area of hemispheric lesion of 31.37 ± 13.32% (MCAO group). After 14 days of EO treatment (MCAO + EO group), the mean area affected was reduced to values 15.02 ± 5.39% (*p* = 0.0217). No lesions were observed in any of the animals of the two control groups (Figure 3).

**Figure 4:**
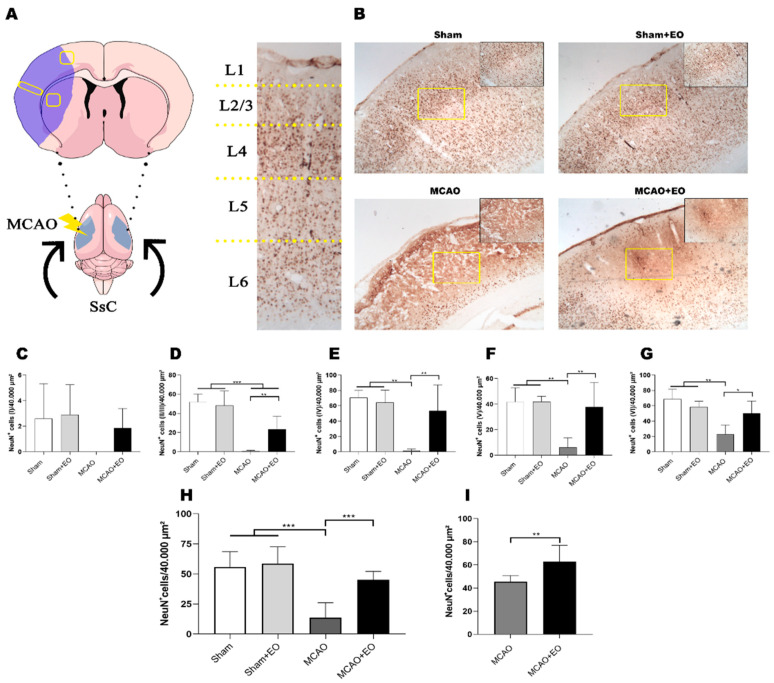
Neuronal death attenuated by the clarified *Euterpe oleracea* extract following middle cerebral artery occlusion (MCAO): (**A**) Brain map adapted from the area analyzed and the cortical layers. (**B**) Representative images of the experimental groups showing the area of cerebral infarction and stained with NeuN antibody. (**C**) Quantitative analysis of the NeuN^+^ cells in layer I. (**D**) Quantitative analysis of the NeuN^+^ cells in layers II/III. (**E**) Quantitative analysis of the NeuN^+^ cells in layer IV. (**F**) Quantitative analysis of the NeuN^+^ cells in layer V. (**G**) Quantitative analysis of the NeuN^+^ cells in layer VI. (**H**) Quantitative analysis of the NeuN^+^ cells in striatum. (**I**) Quantitative analysis of the NeuN^+^ cells in area adjacent to the ischemic lesion (penumbra). The data are expressed as the mean ± SD (*n* = 6 animals per group; C-H, two-way ANOVA followed by Tukey’s post hoc test; I, Student’s *t* test; * *p* < 0.05; ** *p* < 0.01; *** *p* < 0.001).

Cerebral ischemia culminates in cell death, mainly of neurons in the cortical region, which was the reason the NeuN^+^ cell count was conducted per cortical layer (Figure 4A). As no difference was found between the animals of the two control groups (sham and sham + EO), it appears that the administration of the EO extract does not alter the density of the cortical neurons or induce cell death (Figure 4B–G; Supplementary Table S2). 

However, the MCAO did cause neuronal death, as indicated by the significant decrease in the number of NeuN^+^ cells in layers II/III through VI (Figure 4B–G; Supplementary Table S2) of the cortical region of the somatosensory cortex (Figure 3 and Figure 4A). Surprisingly, the animals that received EO extract for 14 days presented significant levels of preservation in the number of NeuN^+^ cells in these same layers (Figure 4B–G; Supplementary Table S2). This result indicates that the EO treatment may activate neuronal survival pathways.

The area adjacent to the ischemic lesion, known as the penumbra, is also of considerable importance, in particular because it is a potentially recoverable area. In the present study, treatment with the EO extract was reflected in a significantly higher number of NeuN^+^ cells in the treated ischemic animals (MCAO + EO group) in comparison with the MCAO group (Figure 4H, Supplementary Table S2; *p* < 0.01). The number of NeuN^+^ cells of the striatum was also reduced significantly in the animals of the MCAO group (Figure 4I, Supplementary Table S2; *p* < 0.01), indicating that the treatment with the EO extract contributed significantly to the preservation of the cells in this region. These findings indicate that the EO treatment offers important protective effects.

### 3.3. Açaí Extract Did Not Cause Biochemical Alterations after Ischemic Stroke

**Figure 5:**
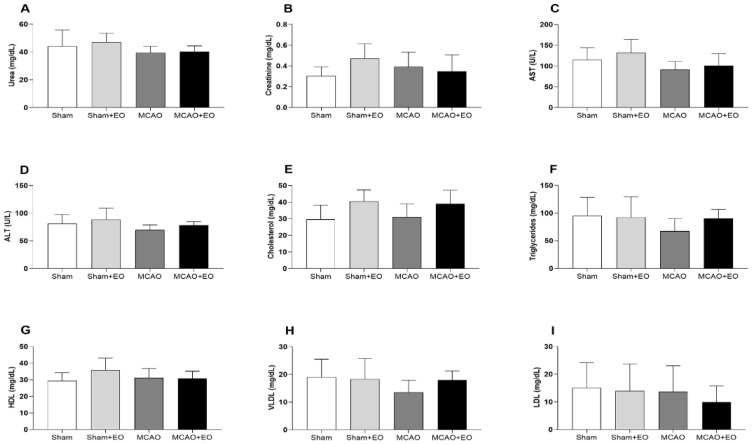
Biochemical analysis of the animals submitted to middle cerebral artery occlusion (MCAO) with clarified *Euterpe oleracea* extract. (**A**) Urea (mg/dL). (**B**) Creatinine (mg/dL). (**C**) AST (aspartate aminotransferase, U/L). (**D**) ALT (alanine aminotransferase, U/L). (**E**) Cholesterol (mg/dL). (**F**) Triglycerides (mg/dL). (**G**) HDL (high-density lipoprotein, mg/dL). (**H**) VLDL (very low-density lipoprotein, mg/dL). (**I**) LDL (low-density lipoprotein, mg/dL). The data are presented as the mean ± SD (*n* = 6–9 animals/group).

No significant variation was found in the biochemical parameters of the animals treated with EO extract following ischemic stroke (Figure 5).

## 4. Discussion

The present study is the first to demonstrate that the extract of *Euterpe oleracea* may attenuate the progression of the damage caused by cerebral ischemia, as demonstrated by the improvement of neurological function, the reduction of ischemic area, and the decrease in neuronal death. We also showed that the application of the extract did not alter either the lipid profile or the liver and kidney functions of the experimental animals. This is an important point because the application does not limit therapy in patients with hepatic or renal impairments.

In most experimental studies, the effects of anthocyanins with antioxidant and anti-inflammatory properties have been shown to attenuate injuries caused by ischemia and reperfusion [33,34,35,36]. The literature elucidates the fact that polyphenols found in EO extract have positive effects on modulating oxidative and inflammatory activity in in vivo and in vitro models by decreasing reactive oxygen species, increasing antioxidant activity, and regulating inflammatory mediators [37,38]. Furthermore, these effects have also been found in humans submitted to ingestion of the EO extract consisting of polyphenolic compounds such as cyanidin-3-glycoside and cyanidin-3-rutinoside, as well as monomeric catechin and epicatechin or oligomeric procyanidins [39,40].

Shin et al. [33] showed that, after 24 h of reperfusion, pretreatment with anthocyanins extracted from the bilberry (300 mg/kg of anthocyanins) enabled a reduction in the size of the cerebral infarction, a finding which may have been related to the suppression of the JNK and p53 signaling pathways. Cui et al. [35] demonstrated that the pretreatment of mice with *Myrica rubra*, whose principal anthocyanin is cyanidin-3-O-glucoside, for seven days prior to the MCAO, was effective for the reduction of both damage to neurological function and the volume of the cerebral infarction, principally in the middle- and high-dose groups (150 and 300 mg/kg).

The neurological scores of the ischemic animals treated with EO extract improved significantly from the ninth day onward. Sunil et al. [34] found that the total oligomeric flavonoids of *Cyperus rotundus* (200 mg/kg, by oral gavage), a traditional Indian herb from the Ayurvedic medicine system, when administered to rats once a day for one week (including four days prior to the MCAO and three days afterward), alleviated the neurological deficit significantly in comparison with untreated ischemic rats. Fu et al. [36] also showed that a high dose (250 or 500 mg/kg) of proanthocyanidins extracted from grape seed improved the neurological score and decreased the area of infarction in comparison with the untreated MCAO group.

Based on this evidence, our findings indicate that both the behavioral improvement and the reduction of the area of cerebral infarction may be related to the presence of anthocyanins (primarily cyanidin-3-glucoside and cyanidin-3-rutinoside) and non-anthocyanin flavonoids (homoorientin and orientin), which are the principal compounds of the clarified EO extract applied in the present study [14,20,25,26]. We also showed that the ischemic animals presented an important motor deficit and that the therapy with EO extract reduced this dysfunction, in particular after one week of treatment. The neurological deficits exhibited by the animals in the present study correlated with the brain region affected by the MCAO which caused injuries in areas that are important for motor processing, such as the somatosensory cortex and the striatum [32]. We also provided the first evidence that the ingestion of the EO extract at a dose of 200 mg/kg/day reduced the area of infarction and contained the neuronal damage. However, one of the limitations of the present study is the lack of evaluation of other motor parameters affected by stroke, such as balance and walking. Thus, more behavioral tests are needed to measure the potential benefit of EO extract in clinical improvement.

Sensorial information is known to have a strong influence on motor processing and is essential for rehabilitation, especially following ischemic stroke [41,42]. Previous studies have shown that changes in the connections between the motor and somatosensory cortexes occur primarily during the acute phase of the stroke. This finding is extremely important because therapies initiated in the acute phase present greater benefits than those initiated later on [43,44,45].

Some studies have also demonstrated that the repair of neuronal connections following ischemic stroke depends on the cellular tissue adjacent to the infarcted area and on other factors, such as glial activation [46,47]. It is also important to note that the motor cortex is able to receive signals directly from the somatosensory cortex, although we cannot rule out the possibility that connections are mediated through the thalamus, especially its posterior medial portion [32,48].

In studies of rodents, these reciprocal connections maintained between the somatosensory and motor cortexes are innervated primarily by cortical layers II/III and V [49,50]. The present study showed a significant level of preservation of the neurons in these layers in the ischemic animals treated with EO extract, indicating that this neuroprotection may be related to the improvement in behavior. From this perspective, the partial integrity of these cortical layers may be important for the maintenance of the afferents/efferents connecting the subcortical and neocortical regions, such as the basal ganglia and the thalamic control centers [51]. This relationship reinforces the importance of the cortical neuroprotection that appeared to be provided by the use of the EO extract.

One other important finding of the present study is that the use of EO extract for 14 days did not appear to alter the lipid homeostasis or metabolism and excretion pathways, such as liver and kidney functions. A number of previous studies have provided evidence of the significant therapeutic potential of compounds extracted from açaí for the control of the lipid profile and improvement of atherosclerosis, in addition to the prevention of hepatic steatosis, although this potential is dose-dependent and also affected by the treatment time [52,53]. 

**Figure 6:**
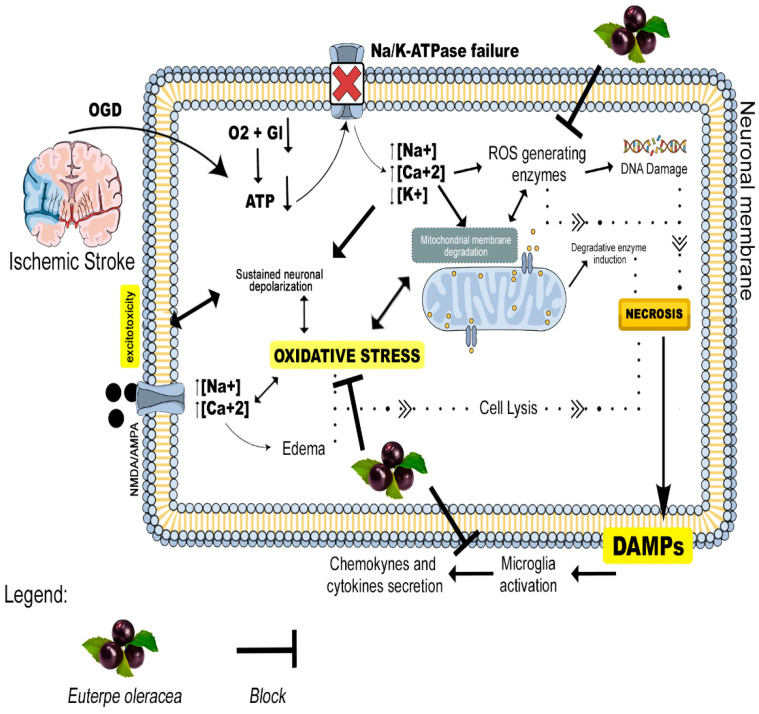
Pathophysiology of ischemic stroke and possible therapeutic targets of the *Euterpe oleracea* extract. OGD = Oxygen and Glucose Deprivation; Gl = Glucose; ATP = Adenosine Triphosphate; ROS = Reactive Oxygen Species; DAMPs = Damage Associated Molecular Patterns; NMDA = N-methyl-D-aspartate; AMPA = Alpha-amino-3-hydroxy-5-methyl-4-isoxazolepropionic Acid.

It is nevertheless important to note that the pathophysiology of ischemic stroke-induced brain lesions is complex and multifactorial. The blockage of blood flow in the principal artery has a rapid effect on the neurons, which die by necrosis forming the nucleus of the ischemia (core of the lesion). However, the peripheral neurons may be supplied by arteries peripheral to the lesion and may undergo late apoptosis if no neuroprotective therapy is implemented [54]. Acute arterial occlusion leads to bioenergetic failure which occurs through oxygen and glucose deprivation (OGD), the loss of ionic homeostasis, excitotoxicity, mitochondrial dysfunction, and the generation of reactive oxygen species (ROS) in the neurons (Figure 6). This process may also lead to the activation of inflammatory cells that release cytokines, leading to an increase in inflammatory processes that may also be responsible for late apoptosis and the expansion of the area of the lesion [54,55]. Stroke triggers cell death by necrosis (acute) and apoptosis/neurodegeneration (late). More studies are therefore needed to better characterize signaling pathways where EO extract can act in the control and attenuation of apoptotic processes, as well as the antioxidant pathways that may be activated and result in neuronal survival.

As observed in previous studies, the clarified EO extract is rich in these compounds [14,20,25,26]. Given this fact, one of the probable therapeutic targets of the EO extract would likely be the reduction in the formation of reactive oxygen species, helping to avoid DNA damage in addition to reducing the oxidative stress caused by glutamate excitotoxicity and mitochondrial damage. The process would improve the inflammatory status through the control of the reactive glia. This is one of the possible hypotheses that may explain the relief of stroke damage by the EO extract (Figure 6). From this perspective, studies involving reactive oxygen species generation, glial reactivity, and inflammatory biomarkers are needed to understand the mechanisms modulated by EO extract compounds on inflammatory and oxidative activity in a model of cerebral ischemia.

## 5. Conclusions

Overall, the findings of the present study indicate clearly that treatment with EO extract in the acute phase following a stroke may trigger signaling pathways that culminate in neuronal survival and contribute to a partial recovery of the clinical scores of the animals. Even so, more systematic studies of the intracellular signaling pathways involved in this process will be needed to better understand its mechanisms.

## Data Availability

The raw data supporting the conclusions of this article will be made available by the authors, without undue reservation.

## Supplementary Materials

The following supporting information can be downloaded at: https://www.mdpi.com/article/10.3390/nu15051207/s1, ST1 (Supplementary Table S1)—Neurological/motor deficit scores of animals submitted to middle cerebral artery occlusion (MCAO) and treated with clarified *Euterpe oleracea* (EO) extract. Data are presented as means ± SD (*n* = 6–9 animals per group). * *p* < 0.05 vs. sham and sham + EO. ^#^ *p* < 0.05 vs. MCAO. Different letters above the data points denote significant differences between days (column) (*p* < 0.05). ST2 (Supplementary Table S2)—Neuronal death attenuated by clarified *Euterpe oleracea* (EO) extract after middle cerebral artery occlusion (MCAO). Data are presented as means ± SD (*n* = 6 animals per group).* *p* < 0.05 vs. sham and sham + EO. ^#^ *p* < 0.05 vs. MCAO.

## References

[1] Benjamin E.J., Virani S.S., Callaway C.W., Chamberlain A.M., Chang A.R., Cheng S., Chiuve S.E., Cushman M., Delling F.N., Deo R. (2018). Heart Disease and Stroke Statistics—2018 Update: A Report From the American Heart Association. Circulation.

[2] Leon-Carrion J., Martin-Rodriguez J.F., Damas-Lopez J., Barroso y Martin J.M., Dominguez-Morales M.R. (2009). Delta–alpha ratio correlates with level of recovery after neurorehabilitation in patients with acquired brain injury. Clin. Neurophysiol..

[3] Povlsen G.K., Longden T.A., Bonev A.D., Hill-Eubanks D.C., Nelson M.T. (2016). Uncoupling of neurovascular communication after transient global cerebral ischemia is caused by impaired parenchymal smooth muscle K_ir_ channel function. J. Cereb. Blood Flow Metab..

[4] Khoshnam S.E., Winlow W., Farzaneh M., Farbood Y., Moghaddam H.F. (2017). Pathogenic mechanisms following ischemic stroke. Neurol. Sci..

[5] Wang L., Xiong X., Zhang L., Shen J. (2021). Neurovascular Unit: A critical role in ischemic stroke. CNS Neurosci. Ther..

[6] Roy A., Datta S. (2021). Medicinal Plants against Ischemic Stroke. Curr. Pharm. Biotechnol..

[7] Tao T., Liu M., Chen M., Luo Y., Wang C., Xu T., Jiang Y., Guo Y., Zhang J.H. (2020). Natural medicine in neuroprotection for ischemic stroke: Challenges and prospective. Pharmacol. Ther..

[8] de Oliveira M.d.S.P., Lemos M.A., dos Santos E.O., dos Santos V.F. (2000). Coeficiente de caminhamento entre caracteres agronômicos e a produção de frutos em açaizeiro (*Euterpe oleracea* Mart.). Rev. Bras. Frutic..

[9] Silva M.P., Cunha V.M.B., Sousa S.H.B., Menezes E.G.O., Bezerra P.d.N., de Farias Neto J.T., Filho G.N.R., Araújo M.E., de Carvalho R.N. (2019). Supercritical CO_2_ extraction of lyophilized Açaí (*Euterpe oleracea* Mart.) pulp oil from three municipalities in the state of Pará, Brazil. J. CO_2_ Util..

[10] Udani J.K., Singh B.B., Singh V.J., Barrett M.L. (2011). Effects of Açai (*Euterpe oleracea* Mart.) berry preparation on metabolic parameters in a healthy overweight population: A pilot study. Nutr. J..

[11] Minighin E.C., Anastácio L.R., Melo J.O.F., Labanca R.A. (2020). Açai (*Euterpe oleracea*) e suas contribuições para alcance da ingestão diária aceitável de ácidos graxos essenciais. Res. Soc. Dev..

[12] Aranha L.N., Silva M.G., Uehara S.K., Luiz R.R., Nogueira Neto J.F., Rosa G., Moraes de Oliveira G.M. (2020). Effects of a hypoenergetic diet associated with açaí (*Euterpe oleracea* Mart.) pulp consumption on antioxidant status, oxidative stress and inflammatory biomarkers in overweight, dyslipidemic individuals. Clin. Nutr..

[13] de Almeida Magalhães T.S.S., de Oliveira Macedo P.C., Converti A., Neves de Lima Á.A. (2020). The Use of *Euterpe oleracea* Mart. As a New Perspective for Disease Treatment and Prevention. Biomolecules.

[14] Arrifano G.P.F., Lichtenstein M.P., Souza-Monteiro J.R., Farina M., Rogez H., Carvalho J.C.T., Suñol C., Crespo-López M.E. (2018). Clarified Açaí (*Euterpe oleracea*) Juice as an Anticonvulsant Agent: In Vitro Mechanistic Study of GABAergic Targets. Oxid. Med. Cell. Longev..

[15] Kang J., Thakali K.M., Xie C., Kondo M., Tong Y., Ou B., Jensen G., Medina M.B., Schauss A.G., Wu X. (2012). Bioactivities of açaí (*Euterpe precatoria* Mart.) fruit pulp, superior antioxidant and anti-inflammatory properties to *Euterpe oleracea* Mart. Food Chem..

[16] Poulose S.M., Fisher D.R., Larson J., Bielinski D.F., Rimando A.M., Carey A.N., Schauss A.G., Shukitt-Hale B. (2012). Anthocyanin-rich Açai (*Euterpe oleracea* Mart.) Fruit Pulp Fractions Attenuate Inflammatory Stress Signaling in Mouse Brain BV-2 Microglial Cells. J. Agric. Food Chem..

[17] de Almeida Magalhães T.S.S., de Oliveira Macedo P.C., Kawashima Pacheco S.Y., da Silva S.S., Barbosa E.G., Pereira R.R., Costa R.M.R., Silva Junior J.O.C., da Silva Ferreira M.A., de Almeida J.C. (2020). Development and Evaluation of Antimicrobial and Modulatory Activity of Inclusion Complex of *Euterpe oleracea* Mart Oil and β-Cyclodextrin or HP-β-Cyclodextrin. Int. J. Mol. Sci..

[18] Pacheco-Palencia L.A., Mertens-Talcott S., Talcott S.T. (2008). Chemical Composition, Antioxidant Properties, and Thermal Stability of a Phytochemical Enriched Oil from Açai (*Euterpe oleracea* Mart.). J. Agric. Food Chem..

[19] Favacho H.A.S., Oliveira B.R., Santos K.C., Medeiros B.J.L., Sousa P.J.C., Perazzo F.F., Carvalho J.C.T. (2011). Anti-inflammatory and antinociceptive activities of *Euterpe oleracea* Mart., Arecaceae, oil. Rev. Bras. Farmacogn..

[20] Souza-Monteiro J.R., Hamoy M., Santana-Coelho D., Arrifano G.P.F.F., Paraense R.S.O.O., Costa-Malaquias A., Mendonça J.R., da Silva R.F., Monteiro W.S.C.C., Rogez H. (2015). Anticonvulsant properties of *Euterpe oleracea* in mice. Neurochem. Int..

[21] Gordon A., Cruz A.P.G., Cabral L.M.C., de Freitas S.C., Taxi C.M.A.D., Donangelo C.M., de Andrade Mattietto R., Friedrich M., da Matta V.M., Marx F. (2012). Chemical characterization and evaluation of antioxidant properties of Açaí fruits (*Euterpe oleraceae* Mart.) during ripening. Food Chem..

[22] Garzón G.A., Narváez-Cuenca C.-E., Vincken J.-P., Gruppen H. (2017). Polyphenolic composition and antioxidant activity of açai (*Euterpe oleracea* Mart.) from Colombia. Food Chem..

[23] Carvalho-Peixoto J., Moura M.R.L., Cunha F.A., Lollo P.C.B., Monteiro W.D., de Carvalho L.M.J., Farinatti P.d.T.V. (2015). Consumption of açai (*Euterpe oleracea* Mart.) functional beverage reduces muscle stress and improves effort tolerance in elite athletes: A randomized controlled intervention study. Appl. Physiol. Nutr. Metab..

[24] Pereira J.C., Martins A.B., Rocha M.C.F., Cavalcante Júnior S.M., Feitosa C.M. (2021). Espécies medicinais do Brasil com potencial anti-inflamatório ou antioxidante: Uma revisão. Res. Soc. Dev..

[25] Dias A.L.S., Rozet E., Larondelle Y., Hubert P., Rogez H., Quetin-Leclercq J. (2013). Development and validation of an UHPLC-LTQ-Orbitrap MS method for non-anthocyanin flavonoids quantification in *Euterpe oleracea* juice. Anal. Bioanal. Chem..

[26] Dias A.L.S., Rozet E., Chataigné G., Oliveira A.C., Rabelo C.A.S., Hubert P., Rogez H., Quetin-Leclercq J. (2012). A rapid validated UHPLC–PDA method for anthocyanins quantification from *Euterpe oleracea* fruits. J. Chromatogr. B.

[27] Alessandra-Perini J., Perini J.A., Rodrigues-Baptista K.C., de Moura R.S., Junior A.P., dos Santos T.A., Souza P.J.C., Nasciutti L.E., Machado D.E. (2018). *Euterpe oleracea* extract inhibits tumorigenesis effect of the chemical carcinogen DMBA in breast experimental cancer. BMC Complement. Altern. Med..

[28] de Bem G.F., Costa C.A., Santos I.B., Cristino Cordeiro V.d.S., de Carvalho L.C.R.M., de Souza M.A.V., Soares R.d.A., Sousa P.J.d.C., Ognibene D.T., Resende A.C. (2018). Antidiabetic effect of *Euterpe oleracea* Mart. (açaí) extract and exercise training on high-fat diet and streptozotocin-induced diabetic rats: A positive interaction. PLoS ONE.

[29] Ferreira L.O., Mattos B.G., Jóia de Mello V., Martins-Filho A.J., da Costa E.T., Yamada E.S., Hamoy M., Lopes D.C.F. (2021). Increased Relative Delta Bandpower and Delta Indices Revealed by Continuous qEEG Monitoring in a Rat Model of Ischemia-Reperfusion. Front. Neurol..

[30] Zhang Z., Qin P., Deng Y., Ma Z., Guo H., Guo H., Hou Y., Wang S., Zou W., Sun Y. (2018). The novel estrogenic receptor GPR30 alleviates ischemic injury by inhibiting TLR4-mediated microglial inflammation. J. Neuroinflamm..

[31] Rousselet E., Kriz J., Seidah N.G. (2012). Mouse Model of Intraluminal MCAO: Cerebral Infarct Evaluation by Cresyl Violet Staining. J. Vis. Exp..

[32] Fukui A., Osaki H., Ueta Y., Kobayashi K., Muragaki Y., Kawamata T., Miyata M. (2020). Layer-specific sensory processing impairment in the primary somatosensory cortex after motor cortex infarction. Sci. Rep..

[33] Shin W.-H., Park S.-J., Kim E.-J. (2006). Protective effect of anthocyanins in middle cerebral artery occlusion and reperfusion model of cerebral ischemia in rats. Life Sci..

[34] Sunil A.G., Kesavanarayanan K.S., Kalaivani P., Sathiya S., Ranju V., Priya R.J., Pramila B., Paul F.D.S., Venkhatesh J., Babu C.S. (2011). Total oligomeric flavonoids of Cyperus rotundus ameliorates neurological deficits, excitotoxicity and behavioral alterations induced by cerebral ischemic–reperfusion injury in rats. Brain Res. Bull..

[35] Cui H.-X., Chen J.-H., Li J.-W., Cheng F.-R., Yuan K. (2018). Protection of Anthocyanin from Myrica rubra against Cerebral Ischemia-Reperfusion Injury via Modulation of the TLR4/NF-$κ$B and NLRP3 Pathways. Molecules.

[36] Fu K., Chen L., Hu S., Guo Y., Zhang W., Bai Y. (2021). Grape seed proanthocyanidins attenuate apoptosis in ischemic stroke. Acta Neurol. Belg..

[37] da Silva Cristino Cordeiro V., de Bem G.F., da Costa C.A., Santos I.B., de Carvalho L.C.R.M., Ognibene D.T., da Rocha A.P.M., de Carvalho J.J., de Moura R.S., Resende A.C. (2018). *Euterpe oleracea* Mart. seed extract protects against renal injury in diabetic and spontaneously hypertensive rats: Role of inflammation and oxidative stress. Eur. J. Nutr..

[38] Bonomo L.d.F., Silva D.N., Boasquivis P.F., Paiva F.A., Guerra J.F.d.C., Martins T.A.F., de Jesus Torres Á.G., de Paula I.T.B.R., Caneschi W.L., Jacolot P. (2014). Açaí (*Euterpe oleracea* Mart.) Modulates Oxidative Stress Resistance in Caenorhabditis elegans by Direct and Indirect Mechanisms. PLoS ONE.

[39] Oliveira de Souza M., Barbosa P., Pala D., Ferreira Amaral J., Pinheiro Volp A.C., Nascimento de Freitas R. (2020). A prospective study in women: Açaí (*Euterpe oleracea* Martius) dietary intake affects serum p-selectin, leptin, and visfatin levels. Nutr. Hosp..

[40] Barbosa P.O., Pala D., Silva C.T., de Souza M.O., do Amaral J.F., Vieira R.A.L., Folly G.A.d.F., Volp A.C.P., de Freitas R.N. (2016). Açai (*Euterpe oleracea* Mart.) pulp dietary intake improves cellular antioxidant enzymes and biomarkers of serum in healthy women. Nutrition.

[41] Schabrun S., Hillier S. (2009). Evidence for the retraining of sensation after stroke: A systematic review. Clin. Rehabil..

[42] Rosenkranz K., Rothwell J.C. (2012). Modulation of Proprioceptive Integration in the Motor Cortex Shapes Human Motor Learning. J. Neurosci..

[43] Friel K.M., Barbay S., Frost S.B., Plautz E.J., Hutchinson D.M., Stowe A.M., Dancause N., Zoubina E.V., Quaney B.M., Nudo R.J. (2005). Dissociation of Sensorimotor Deficits After Rostral Versus Caudal Lesions in the Primary Motor Cortex Hand Representation. J. Neurophysiol..

[44] Nudo R.J., Friel K.M., Delia S.W. (2000). Role of sensory deficits in motor impairments after injury to primary motor cortex. Neuropharmacology.

[45] Biernaskie J., Chernenko G., Corbett D. (2004). Efficacy of Rehabilitative Experience Declines with Time after Focal Ischemic Brain Injury. J. Neurosci..

[46] Petreanu L., Mao T., Sternson S.M., Svoboda K. (2009). The subcellular organization of neocortical excitatory connections. Nature.

[47] Minamisawa G., Kwon S.E., Chevée M., Brown S.P., O’Connor D.H. (2018). A Non-canonical Feedback Circuit for Rapid Interactions between Somatosensory Cortices. Cell Rep..

[48] Mo C., Sherman S.M. (2019). A Sensorimotor Pathway via Higher-Order Thalamus. J. Neurosci..

[49] Smith J.B., Alloway K.D. (2013). Rat whisker motor cortex is subdivided into sensory-input and motor-output areas. Front. Neural Circuits.

[50] Mao T., Kusefoglu D., Hooks B.M., Huber D., Petreanu L., Svoboda K. (2011). Long-Range Neuronal Circuits Underlying the Interaction between Sensory and Motor Cortex. Neuron.

[51] Tennant K.A., Adkins D.L., Donlan N.A., Asay A.L., Thomas N., Kleim J.A., Jones T.A. (2011). The Organization of the Forelimb Representation of the C57BL/6 Mouse Motor Cortex as Defined by Intracortical Microstimulation and Cytoarchitecture. Cereb. Cortex.

[52] da Silva R.C., Batista A., da Costa D.C.F., Moura-Nunes N., Koury J.C., da Costa C.A., Resende Â.C., Daleprane J.B. (2018). Açai (*Euterpe oleracea* Mart.) seed flour prevents obesity-induced hepatic steatosis regulating lipid metabolism by increasing cholesterol excretion in high-fat diet-fed mice. Food Res. Int..

[53] Feio C.A., Izar M.C., Ihara S.S., Kasmas S.H., Martins C.M., Feio M.N., Maués L.A., Borges N.C., Moreno R.A., Póvoa R.M. (2012). *Euterpe oleracea* (Açai) Modifies Sterol Metabolism and Attenuates Experimentally-Induced Atherosclerosis. J. Atheroscler. Thromb..

[54] Fann D.Y.-W., Lee S.-Y., Manzanero S., Chunduri P., Sobey C.G., Arumugam T.V. (2013). Pathogenesis of acute stroke and the role of inflammasomes. Ageing Res. Rev..

[55] Brait V.H., Arumugam T.V., Drummond G.R., Sobey C.G. (2012). Importance of T Lymphocytes in Brain Injury, Immunodeficiency, and Recovery after Cerebral Ischemia. J. Cereb. Blood Flow Metab..

