# Supplementary material for: Oral Treatment with the Extract of Euterpe oleracea Mart. Improves Motor Dysfunction and Reduces Brain Injury in Rats Subjected to Ischemic Stroke
Source: Nutrients. 2023 Feb 28;15(5):1207. doi: 10.3390/nu15051207 (PMC10005587; doi:10.3390/nu15051207)
Supplement: Supplementary file 1 [file nutrients-15-01207-s001.zip › nutrients-2154316-supplementary.pdf]

Supplementary 1

ST1 (Supplementary Table 1) – Neurological/motor deficit scores of animals submitted to middle cerebral artery occlusion (MCAO) and treated with clarified *Euterpe oleracea* (EO) extract. Data are presented as means ± SD (n = 6-9 animals per group). * $p < 0.05$: vs Sham and Sham+EO. # $p < 0.05$: vs MCAO. Different letters above the data points denote significant differences between days (column) ($p < 0.05$).

Days	Sham	Sham+EO	MCAO	MCAO+EO
1st Day	0.0 ± 0.0 a	0.0 ± 0.0 a	3.0 ± 0.0 *a	2.5 ± 0.53 *#a
2nd Day	0.0 ± 0.0 a	0.0 ± 0.0 a	2.5 ± 0.54 *a	2.5 ± 0.53 *a
3rd Day	0.0 ± 0.0 a	0.0 ± 0.0 a	2.5 ± 0.54 *a	2.5 ± 0.53 *a
4th Day	0.0 ± 0.0 a	0.0 ± 0.0 a	2.3 ± 0.51 *b	2.25 ± 0.46 *a
5th Day	0.0 ± 0.0 a	0.0 ± 0.0 a	2.3 ± 0.51 *b	2.25 ± 0.46 *a
6th Day	0.0 ± 0.0 a	0.0 ± 0.0 a	2.3 ± 0.51 *b	2.0 ± 0.0 *a
7th Day	0.0 ± 0.0 a	0.0 ± 0.0 a	2.3 ± 0.51 *b	2.0 ± 0.0 *a
8 Day	0.0 ± 0.0 a	0.0 ± 0.0 a	2.3 ± 0.51 *b	2.0 ± 0.0 *a
9th Day	0.0 ± 0.0 a	0.0 ± 0.0 a	2.3 ± 0.51 *b	1.87 ± 0.35 *#b
10th Day	0.0 ± 0.0 a	0.0 ± 0.0 a	2.3 ± 0.51 *b	1.87 ± 0.35 *#b
11th Day	0.0 ± 0.0 a	0.0 ± 0.0 a	2.3 ± 0.51 *b	1.75 ± 0.46 *#b
12th Day	0.0 ± 0.0 a	0.0 ± 0.0 a	2.3 ± 0.51 *b	1.75 ± 0.46 *#b
13th Day	0.0 ± 0.0 a	0.0 ± 0.0 a	2.3 ± 0.51 *b	1.62 ± 0.51 *#b
14th Day	0.0 ± 0.0 a	0.0 ± 0.0 a	2.3 ± 0.51 *b	1.62 ± 0.51 *#b

ST2 (Supplementary Table 2) –Neuronal death attenuated by clarified *Euterpe oleracea* (EO) extract after middle cerebral artery occlusion (MCAO). Data are presented as means ± SD (n = 6 animals per group).* $p < 0.05$: vs Sham and Sham+EO. # $p < 0.05$: vs MCAO.

Region	Sham	Sham+EO	MCAO	MCAO+EO
Layer I	2.59 ± 2.74	2.90 ± 2.33	0.0 ± 0.0	1.85 ± 1.53
Layer II/III	52.06 ± 8.08	48.18 ± 15.06	0.46 ± 0.93*	23.46 ± 13.61*#
Layer IV	70.53 ± 9.55	64.00 ± 16.44	1.53 ± 2.01*	53.23 ± 33.90#
Layer V	41.78 ± 10.95	41.88 ± 4.26	6.06 ± 7.60*	37.79 ± 19.13#
Layer VI	68.78 ± 12.84	58.50 ± 7.36	22.91 ± 11.98*	50.32 ± 15.72#
Striatum	55.75 ± 12.74	58.50 ± 14.00	13.67 ± 12.38*	45.06 ± 7.07#
Penumbra	-	-	45.42 ± 5.32	62.59 ± 14.31#
